# 

**Published:** 1888-04-07

**Authors:** 


					PRICE ONE PENNY. w
Snnnm da A/I
No. 80, Vol IV.-
April 7th, 1888.
fer Annum, es. ea.. post nee.
[nil JajQ\ Monthly Parts, 6d.; post free, 7la,
(Registered at the General Post Office WL <m> <?> u?vu*1"' ou" ,5U'
as a Newspaper.] Ditto per Ann., 7s. 6d. post free.

				

